# Incidental Discovery of Hepatocellular Carcinoma on 18F-PSMA PET CT Performed for Prostate Cancer Reassessment

**DOI:** 10.1155/2023/1458175

**Published:** 2023-12-13

**Authors:** Lauren Hekman, Eva Napierkowski, Natalie C. Hartman, Jeffrey L. Ellis, Robert H. Wagner, Davide Bova, Maria M. Picken, Robert C. Flanigan

**Affiliations:** ^1^Loyola University Medical Center, Department of Urology, USA; ^2^Loyola University Medical Center, Department of Nuclear Medicine, USA; ^3^Loyola University Medical Center, Department of Radiology, USA; ^4^Loyola University Medical Center, Department of Pathology, USA

## Abstract

Prostate-specific membrane antigen positron emission tomography (PSMA PET) has been approved by the Food and Drug Administration (FDA) to identify prostate cancer in the setting of biochemical recurrence but can also identify other malignancies. 18F-PSMA PET has not been studied as a potential tool for hepatocellular carcinoma (HCC). We describe the case of a 76-year-old male with a rising prostate-specific antigen (PSA) after definitive prostate cancer treatment and no prior liver pathology who was incidentally found to have HCC on 18F-PSMA PET.

## 1. Introduction

PSMA is a misnomer, as this membrane protein is not specific to prostate cells. PSMA expression has been identified in both benign extraprostatic tissues (salivary and parotid glands, renal tubules, small intestine, and the Schwann cells of the central nervous system) and certain malignancies, including HCC, renal cell carcinoma (RCC), esophageal cancer, and lung cancer [1–4]. Histologic examination of HCC tissue has demonstrated marked PSMA staining of tumoral neovasculature in a previous study [5]. PSMA PET is primarily used as a diagnostic tool for the detection of recurrent prostate cancer, but its ability to detect extraprostatic malignancies presents a potential new diagnostic avenue for cancers like HCC.

HCC is not uniformly identifiable on all types of PET CT. HCC is inconsistently visible on 18F-fluorodeoxyglucose (FDG) PET but better detected using 68Ga-PSMA PET [6]. Imaging characteristics of HCC on 18F-PSMA PET are not well delineated, nor has the modality's ability to consistently detect HCC been established. Only one prior case report described a patient with polymetastatic prostate cancer and a hypermetabolic liver lesion ultimately found to be HCC on 18F-PSMA PET [1]. Our case is the second in existing literature to describe HCC identification on 18F-PSMA PET ordered for evaluation of extrahepatic pathology.

## 2. Case Presentation

This is a 76-year-old male with a PSA rise to 1.8 ng/mL eight years after completing proton therapy (nadir PSA 0 ng/mL) for low-risk prostate cancer. His previous urologic history included bilateral RCC, and he had undergone left partial nephrectomy and right radical nephrectomy approximately 25 years prior, with no evidence of disease on subsequent surveillance. He had no known prior liver pathology.

**Figure 1:**
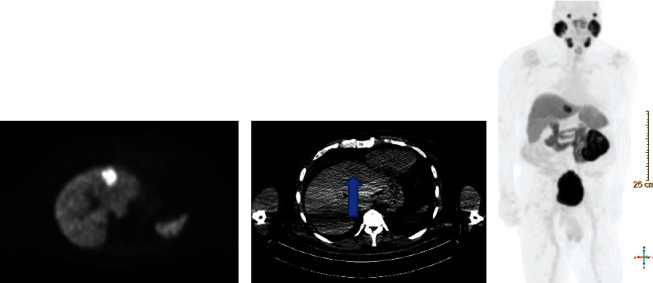
(a–c) PET-CT with 18F-PSMA. Axial (a) and 3D (c) PET images demonstrate a large focus of markedly and heterogeneously increased radiotracer uptake in the left lobe of the liver. Unenhanced low-dose CT image at the corresponding level with narrow window demonstrates a subtle hypodense ill-defined nodule anteriorly in the left hepatic lobe (arrow).

**Figure 2:**
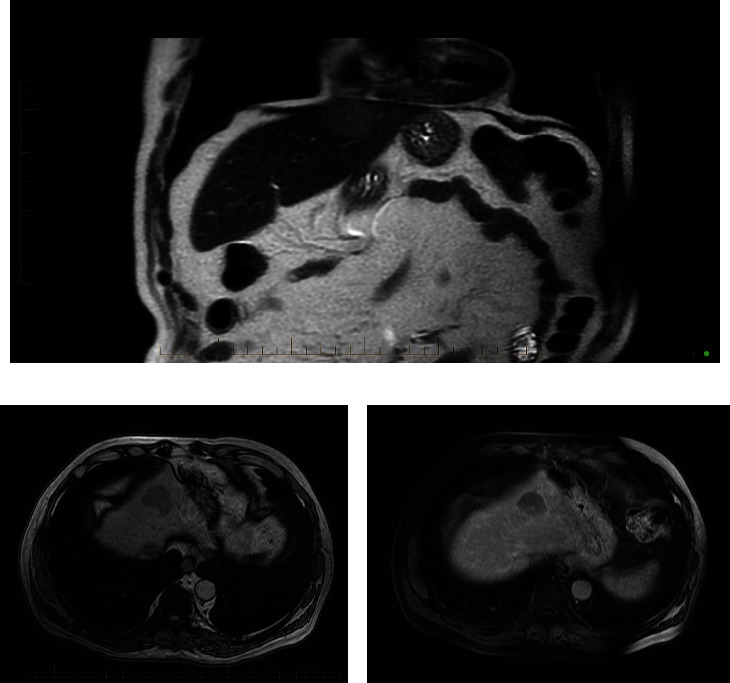
(a–c) MRI of the abdomen. Coronal T2 HASTE image (a) demonstrates a round focus of subtly increased T2 signal in the anterior left hepatic lobe (arrow). Axial gradient echo opposite phase image (b) demonstrates a round focus of decreased signal in the anterior left hepatic lobe (arrow). Axial fat-suppressed T1 image (c) shows the lesion with portal venous washout represented by decreased enhancement in comparison to the surrounding healthy hepatic parenchyma, a feature suspicious for malignancy. Of note, this hepatocellular carcinoma did not demonstrate the more typical arterial enhancement.

He underwent an 18F-PSMA PET CT for prostate cancer restaging. PSMA PET noted two tracer-avid lesions: one in the midprostate posterolateral peripheral zone and one in the left lobe of the liver. The prostate abnormality was only mildly PET-avid, with a standardized uptake value (SUV) of 3.6. The liver lesion had an SUV max of 14.1, well above the physiologic uptake of the liver (SUV max 6.3). Despite being clearly visible on the PET portion of the study, corresponding CT images of the liver only showed a subtle, ill-defined abnormality in the left hepatic lobe (Figure 1). A kidney/adrenal MRI study obtained to better characterize this hepatic lesion and to rule out RCC recurrence demonstrated an area of increased T2 signal in the liver corresponding to the PSMA PET abnormality without other pathologic intra-abdominal findings (Figure 2).

**Figure 3:**
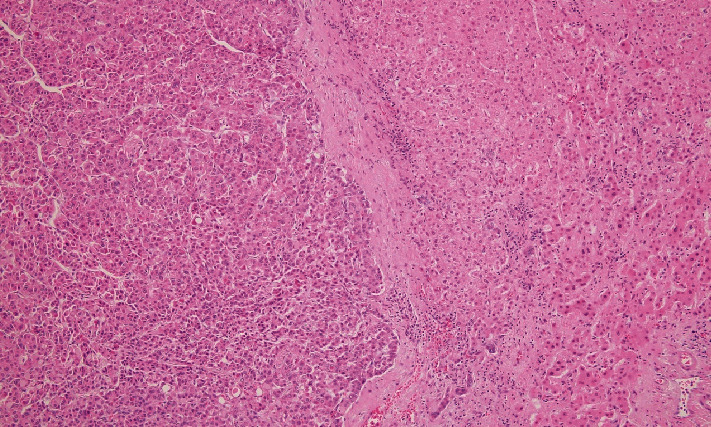
Moderately differentiated hepatocellular carcinoma is seen on the left while the adjacent uninvolved liver parenchyma is unremarkable. PSMA immunohistochemistry was positive only in tumor sinusoid and negative in the surrounding tissue H&E stain, original magnification 100x.

**Figure 4:**
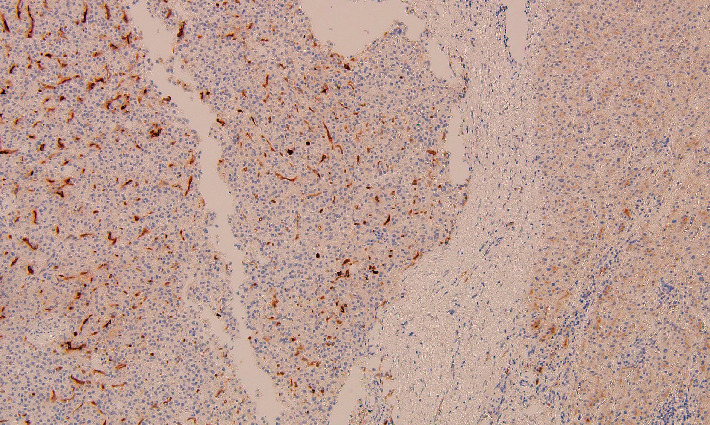
Immunoperoxidase stain for PSMA highlights diffusely positive stain on sinusoidal endothelial cells in the tumor while the uninvolved liver parenchyma is negative. Original magnification 100x.

The patient underwent a percutaneous liver biopsy, and pathologic examination revealed moderately differentiated hepatocellular carcinoma. Alpha fetoprotein (AFP) level, hepatitis serologies, and liver function tests were within normal limits. He then underwent a laparoscopic left lateral segmentectomy of the liver. Pathology examination confirmed the diagnosis of moderately differentiated hepatocellular carcinoma in the setting of a noncirrhotic liver (Figure 3). Immunostain for PSMA demonstrated diffusely highlighted sinusoidal endothelial cells (Figure 4). PSMA immunohistochemistry was positive only in tumor sinusoid and negative in the surrounding tissue. Given his three malignancies (including bilateral RCC), this patient was referred to a genetic counselor and is undergoing testing for hereditary syndromes.

## 3. Discussion

Suboptimal HCC detection with alternative radiotracers has generated interest in capitalizing on extraprostatic PSMA expression and exploring PSMA PET's utility in HCC. 18F-FDG PET was previously trialed as a potential diagnostic modality for HCC but was found to have suboptimal HCC sensitivity (<50%) [7]. In three recent pilot studies and one case report, patients being evaluated for HCC underwent both 18F-FDG PET and 68Ga-PSMA PET to compare diagnostic accuracy. 68Ga-PSMA PET demonstrated consistent intense tracer uptake in HCC lesions, with a substantial proportion of these lesions going undetected on 18F-FDG PET [5–9]. A 2022 prospective study on 68Ga-PSMA PET noted more pronounced metabolic activity within HCC lesions of higher tumor grade [10].

While there is abundant emerging literature evaluating the 68Ga-PSMA radiotracer in HCC diagnostics, little has been published about alternative radiopharmaceutical use in HCC imaging, such as 18F-PSMA. 18F-PSMA's longer half-life has made it an attractive tool for assessing prostatic/pelvic lesions, but there are few reports of using this tracer for HCC workup. Once case report by Zhao et al. described a patient with polymetastatic prostate cancer and an elevated AFP who underwent a 18F-PSMA PET for prostate cancer restaging [1]. He was found to have PET-avid lesions in the prostate, axial skeleton, and liver. The liver lesion which was confirmed as HCC on biopsy demonstrated marked metabolic activity (SUV max 27.5), with more modest PET avidity in the prostate (SUV max 11.7). While our patient did not demonstrate metastatic disease, our case did share this pattern of pronounced tracer uptake in the HCC lesion and more muted PET avidity in prostatic foci. Further investigation is needed to characterize typical SUV values for HCC sites and determine if similar uptake differentials are typical of a primary liver malignancy. Based on our experience and the case report by Zhao et al., it is our recommendation that hepatic lesions identified on 18F-PSMA PET should undergo a liver biopsy.

HCC can be diagnosed with noninvasive imaging if contrast-enhanced CT or MRI exhibit the neoplasm's signature arterial hyperenhancement and subsequent portal/venous washout pattern [11]. Lesions without washout tend to represent other pathology, such as arterioportal shunts [11]. However, in cases with only noncontrast imaging, hepatic lesions may be less readily identifiable. Our patient had a noncontrast CT as part of his PSMA PET; on this study, his hepatic lesion was a subtle hypodense region (radiodensity 30 Hounsfield units, compared to 62 Hounsfield units in surrounding liver parenchyma) and difficult to identify as a distinct abnormality. His MR study did have contrast images, but the study was a dedicated renal exam, and most series did not visualize the liver lesion. PSMA PET may function as a helpful diagnostic tool for situations where contrast administration is not feasible and lesions are not grossly apparent without enhancement (prohibitive renal function, severe contrast allergy, etc.).

An unusual component of our patient's case is his three primary malignancies. He had a remote history of bilateral RCC and had undergone a right radical nephrectomy followed by a left partial nephrectomy roughly 25 years prior to his HCC diagnosis (exact pathology not available). His maternal family history includes a sister with unilateral renal cell carcinoma identified in her 60s, a mother with gynecological cancer, and a maternal aunt and two maternal cousins diagnosed with breast cancer in their 50s. His father was diagnosed with prostate cancer at 81 years of age, as well as a paternal cousin at 64 years of age. He is currently undergoing genetic testing. BRCA germline mutations are associated with a higher propensity for cholangiocarcinoma, but studies have not established a strong link between BRCA1/BRCA2 mutations and HCC [12, 13].

PSMA expression in HCC may eventually unlock additional treatment options for this malignancy. A phase II clinical trial examining 18F-PSMA PET's ability to accurately assess HCC tumor sites and treatment responses is currently enrolling patients [14]. PSMA has also been suggested as a potential cellular target for lesion-directed radionuclide therapies, and PSMA PET imaging may offer a more accurate roadmap for local ablation techniques [10].

## 4. Conclusions

Previous studies have established that HCC is well-identified on 68Ga-PSMA PET. The diagnostic accuracy of 18F-PSMA PET for HCC has not been studied; only one other case report in the literature describes 18F-PSMA PET identifying a primary liver malignancy. Limited existing data on 18F-PSMA PET suggests that hepatic lesions visualized with this modality should undergo liver biopsy to evaluate for HCC. Ongoing trials are examining if 18F-PSMA can function as an accurate diagnostic tool for HCC. On the therapeutic front, PSMA expression in HCC tumor neovasculature may eventually provide an avenue for lesion-directed radiopharmaceuticals or other targeted interventions.

## Data Availability

Data is available upon request.
